# Posterior Vertebral Body Tethering: A Preliminary Study of a New Technique to Correct Lenke 5C Lumbar Curves in Adolescent Idiopathic Scoliosis

**DOI:** 10.3390/children11020157

**Published:** 2024-01-26

**Authors:** Jean-Damien Metaizeau, Delphy Denis

**Affiliations:** Department of Paediatric Orthopaedic Surgery, Chu Dijon, 21000 Dijon, France

**Keywords:** growth modulation, idiopathic scoliosis

## Abstract

Vertebral body tethering has been approved for adolescent scoliosis correction. The usual approach is anterior, which is relatively easy for the thoracic spine, but becomes much more challenging for the lumbar curves, with a higher rate of complications. The purpose of this study was to describe and evaluate the first results of a new posterior vertebral body tethering (PVBT) technique using pedicle screws through a posterolateral Wiltse approach. Twenty-two patients with 5C idiopathic scoliosis (Lenke classification) were included in this retrospective study, with a follow up of 2 years after surgery. The lumbar and thoracic curves were measured pre-operatively (POS), at first standing (FS) and at 2 years (2Y). Complications were also analysed. A significant improvement of 30.7° was observed for lumbar curve magnitude between POS and 2Y. Both the thoracic kyphosis and the lumbar lordosis remained stable. Thirteen complications were noted: three led to posterior arthrodesis, three needed a revision with a good outcome, and the seven others (overcorrections, screw breakage or pull-out) achieved a good result. PVBT seems an effective technique for the management of type 5 C adolescent idiopathic scoliosis. The complication rate seems high but is probably secondary to the learning curve of this new technic as it concerns only the first half of the patients.

## 1. Introduction

Scoliosis affects thousands of children worldwide. A curve of 45 degrees or higher is typically regarded as an indication to surgical treatment, as these curves typically continue to progress even in skeletally mature patients [1,2,3,4]. While various treatment options exist to address this condition, one innovative technique has gained increasing attention in recent years: Anterior Vertebral Body Tethering (AVBT). This surgical technique was developed for the treatment of severe scoliosis in adolescents with two main objectives: to avoid fusion and maintain spine flexibility [5,6,7,8]. 

It is an alternative option to Posterior Spinal Fusion (PSF), which remains the gold standard as it provides sustainable long-term outcomes, but is associated with potential long-term complications such as degenerative disc disease, back pain, radiculopathy and loss of mobility [2,9,10,11].

Most of the studies on anterior vertebral body tethering focus on the thoracic spine; there are very few for lumbar curves and to our knowledge, none with a posterior approach. The lumbar spine is the most mobile part of the spine, so to maintain its mobility is essential. But in these cases, surgery is more complex as a mini lumbar approach is needed; this is technically demanding, with potential complications [5,12]. Indeed, it is more difficult to put the screws in the lumbar area through the ilio-psoas muscle between nerves and vessels than in the thoracic spine, and a lot of surgeons are not used to these anterior approaches even though they are very familiar with posterior approaches.

This is why we developed the Posterior Vertebral Body Tethering (PVBT), using the same principles; brace effect and growth modulation [1], but through a posterior Wiltse approach [13]. This makes the technique easier, with the benefit of placing the screws posteriorly, avoiding anterior screws, which could lead to a loss of lordosis. 

The main aim of the study was to verify that posterior vertebral body tethering is effective in the correction of a major curve. We also wanted to evaluate the behaviour of the thoracic curves, the modifications in the sagittal plane and the complications.

## 2. Materials and Methods

The present retrospective study was performed between 2018 and 2022 in our institution by two senior surgeons. All families received an information letter.

### 2.1. Patient Selection

The inclusion criteria were:

- Diagnosis of idiopathic scoliosis from 11 to 16 years old;
- Severe progressive curves: >35°;
- Type 5C on the Lenke Classification;
- Surgical treatment using a “Posterior Vertebral Body Tethering” as described in the operative technique;
- A minimum follow-up of 2 years.

The exclusion criteria were:

- Curves other than Lenke 5C;
- Curves < 35° or >60°;
- Secondary scoliosis.

### 2.2. Surgical Technique

Under general anaesthesia, the patient is placed in a prone position with all support areas padded.

**Figure 1:**
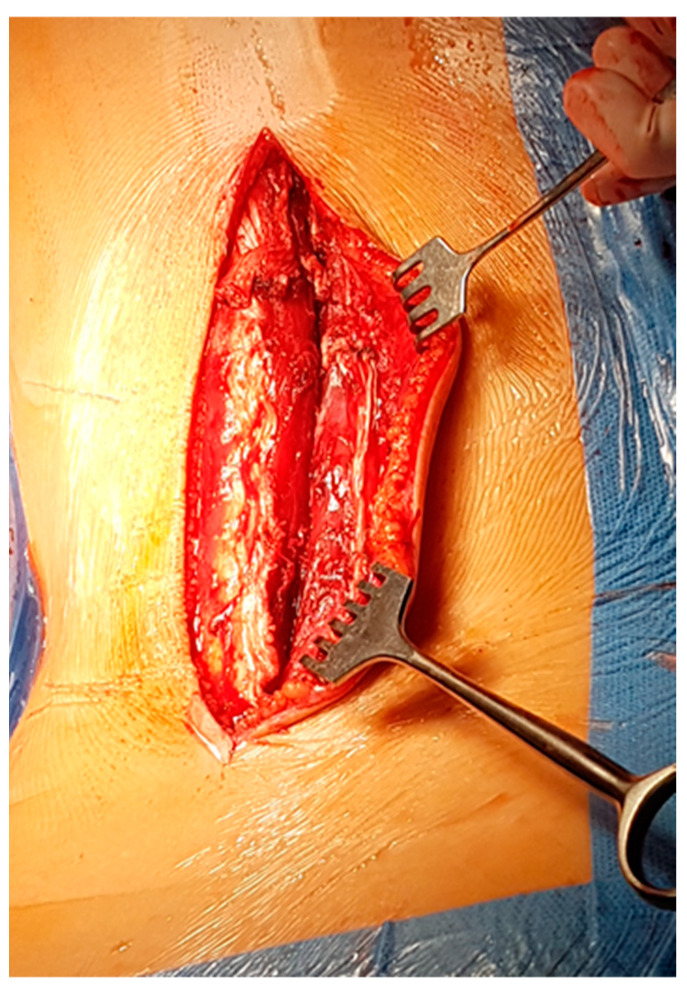
View of the space between the lateral longissimus and medial multifidus muscles.

**Figure 2:**
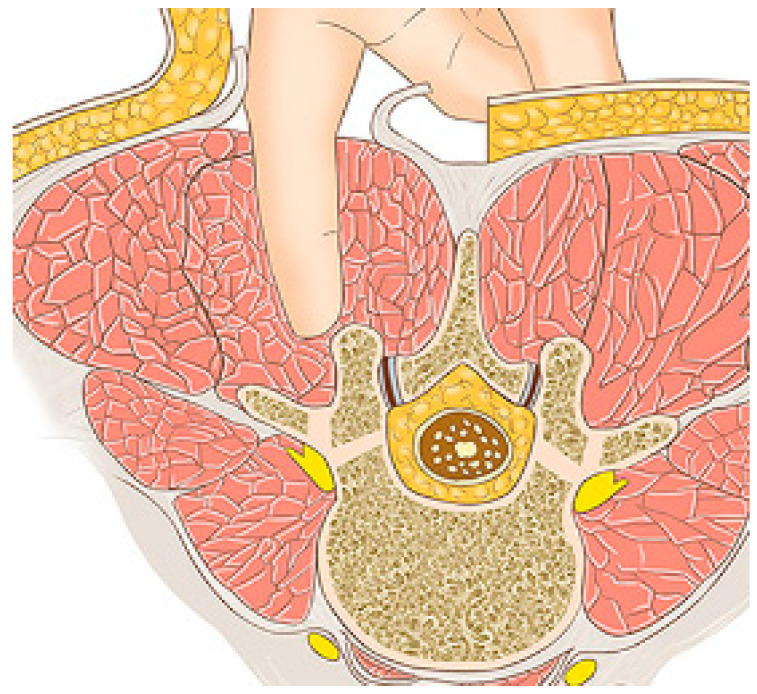
The space between the muscles (Ref. Ying-jie Lu, Orthopaedic Surgery [15]); it allows easy access to the joint and the transverse process.

A Wiltse approach is used [13,14,15]. A midline skin incision is made, and the superficial and deep fasciae are opened longitudinally, approximately 2–3 cm laterally on the convex side. A blunt separation of the medial multifudus and the lateral longissimus is made with the fingers (Figure 1 and Figure 2).

**Figure 3:**
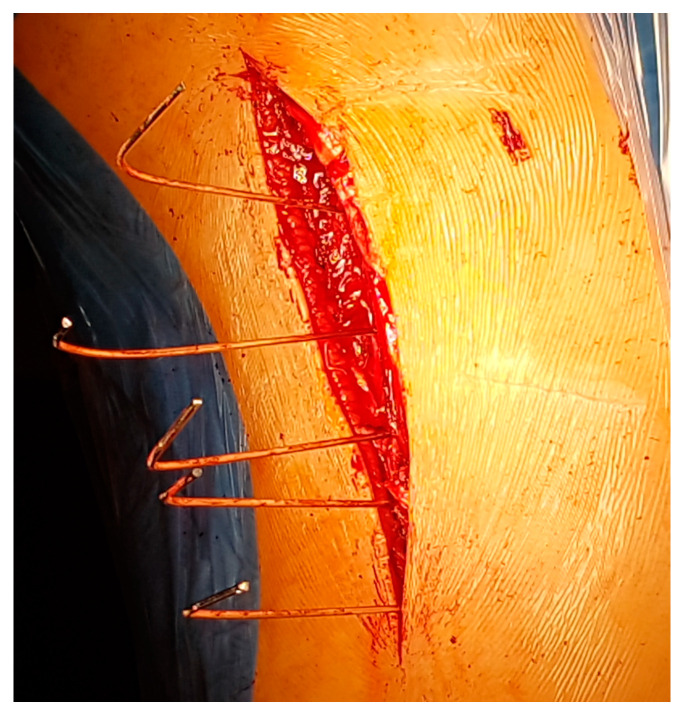
View of the pins: they are stuck to the theoretical entry point and bent for a better identification on the X-ray.

**Figure 4:**
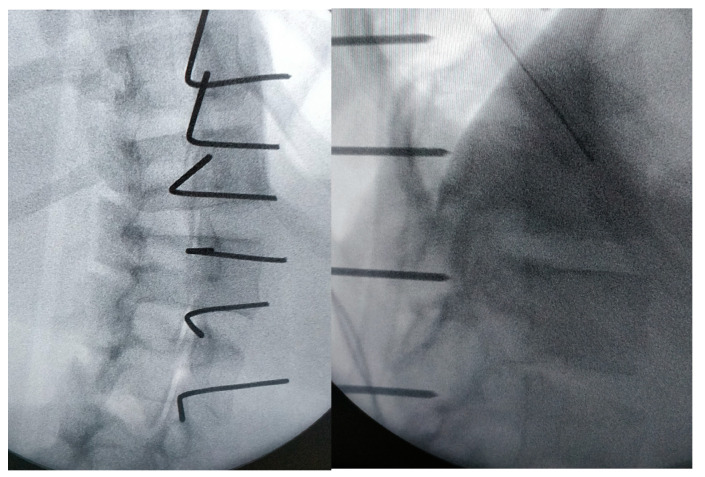
Frontal and sagittal view on the X-ray: it allows the perfect entry point and the right direction of the screws to be checked.

This makes it possible to identify the transverse process and joint of each vertebra. K wire is stuck to the theoretical entry point of the screws at each level under fluoroscopy (Figure 3 and Figure 4); note they are bent at 90° to better identify their position on the X-ray. 

**Figure 5:**
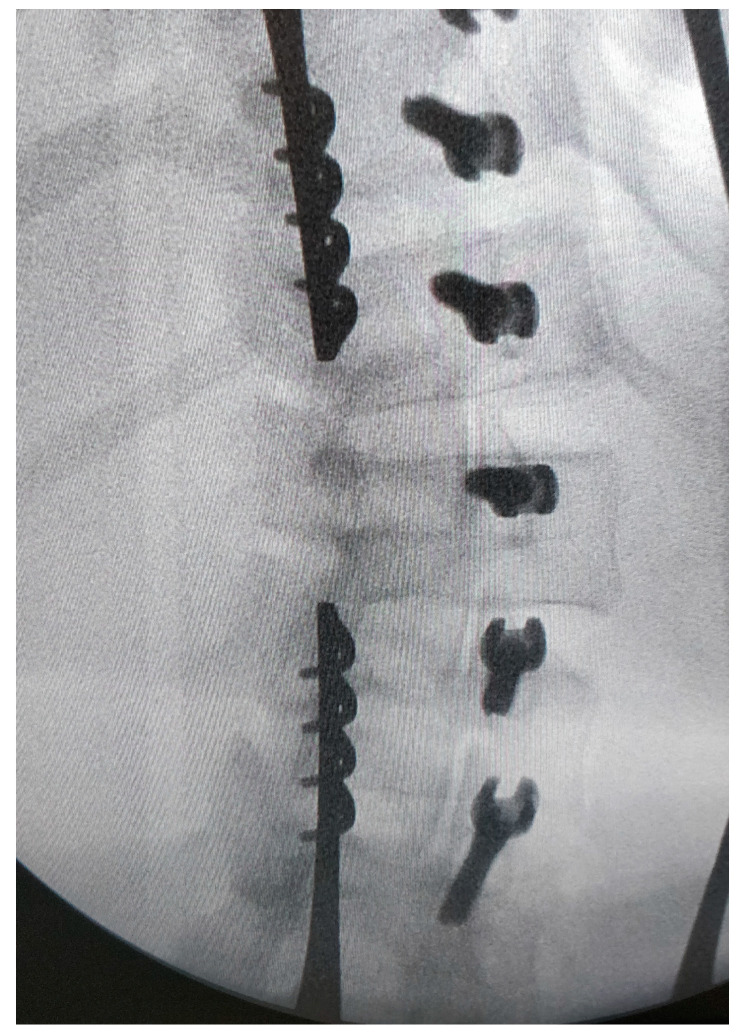
Frontal view of the screws; to check their good position, a sagittal view is also performed.

Then, if possible, a three-dimensional acquisition is made to evaluate the ideal path of the screws for each level. A Pediguard^®^ is used to enter the pedicles safely and avoid the wrong way as much as possible. With a palpator, the presence of bone all around the tunnel is checked, allowing the length to also be measured. The screws (diameter 5.5 to 6.5 mm) are then put in place in the pedicles. Of course, for this step, surgeons should use the same technique they usually use for pedicle screws. Then, new fluoroscopy is performed to assess their perfect position (Figure 5).

For those who have access, the same procedure can be carried out under navigation. 

**Figure 6:**
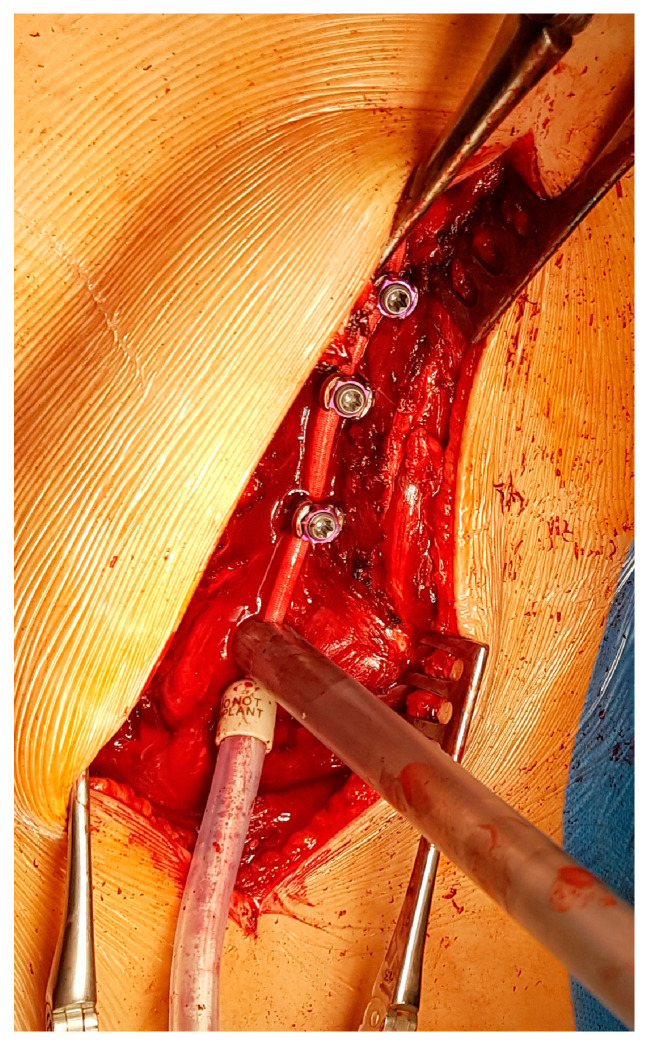
Tightening of the cord: the device is placed against the screws to put tension in the cable, and then the bolt is tightened. The procedure is repeated for each level.

The chord is progressively placed within the screw heads from the cranial to the caudal end. Curve correction is performed with a combination of external manoeuvres (push on the convex side) and tension of the tether level by level using the appropriate tool (Figure 6). 

Both fasciae are closed, the superficial fascia with the subcutaneous tissue, and then the skin with an intradermic suture. Patients walk at day 1 and are usually discharged at day 2 or 3.

The full spine when erect and the bending X-rays help to implement the right levels. The highest cranial level was T10 and the most caudal L5.

Three different types of materials were used: the CTJ+™ from NEUROFRANCE Implants^®^ (La Ville aux Clercs, France), the BRAIVE™ from MEDTRONIC^®^ (Minneapolis, MN, USA) and the Reflect™ from GLOBUS Medical^®^ (Audubon, PA, USA).

### 2.3. Post-Operative Management

The first full-spine erect radiograph is performed at day 2 or 3, then at 1.5, 6, 12, 18 and 24 months post-op, and then once a year. 

Sport is authorized after 6 weeks if the patients feel confident. There was no brace after surgery.

The device removal is not planned systematically, but has been carried out in some cases.

### 2.4. Outcomes of Interest

Baseline demographic data such as gender, age and Risser grade at surgery date were collected.

The major curve (instrumented) and compensatory curves were measured using the Cobb method, and pre-operative standing (POS), pre-operative bending for the major curve (POB), at first standing (FS) and at two years (2Y). We also evaluated thoracic kyphosis and lumbar lordosis.

The duration of hospitalisation, operative time and all the complications were recorded.

### 2.5. Statistical Analysis

The statistical analysis was performed with the software “BiostaTGV” (www.biostatgv.sentiweb.fr). Continuous data were expressed as mean and standard deviation, while the categorical variables were expressed as percentages. A two-sided paired t-test was performed to compare the different radiographic data. A 95% confidence interval was set for all comparisons (*p* = 0.05).

## 3. Results

### 3.1. Patient Selection and Demographic Data

During the observation period, 22 patients (16 girls and 6 boys) meeting the inclusion criteria were treated with posterior vertebral body tethering in our institution. 

The mean age was 14 years old (12 to 16) and mean weight was 49 kg (35 to 64) with a Risser index of 1.5 (0 to 3). 

Hospitalization stay was 3.1 days (2 to 5) and surgery time was 118 min (88 to 172).

The other data are summarized in Table 1 and Table 2.

Both the major and secondary curves corrected significantly between pre-operative standing and two years: 30.7° (*p* = 0.00005) and 9.2° (*p* = 0.0013), respectively. In fact, we found the same results when comparing pre-operative standing and first standing: 23.6° (*p* = 0.00000002) for the major curve and 7.2° (*p* = 0.000102) for the secondary curve. But there was no significant difference between first standing and two years: respectively, 7.1° (*p* = 0.79) and 2° (*p* = 0.86).

The conclusion is the same when comparing pre-operative bending and first standing for the major curve: 4.7° (*p* = 0.96).

In the sagittal plane, the thoracic and lumbar curves did not significantly change between pre-operative standing (23.2° and 41.7°), first standing (25.1° and 42.5°) and two years (26.8° and 42.8°) (*p* always > 0.5).

### 3.2. Complications

All the complications observed and the treatments are summarised in Table 3.

Pain was considered a complication when not usual after spine surgery. One patient needed painkillers and Gabapentine^®^; the pain decreased with time, allowing the drugs to be stopped. The second had a typical nervous irritation which led to a CT-scan which showed an intra-canular screw in L1; the removal of the screw resolved the issue without compromising the correction. The third one had persistent pain, which did not require painkillers but was annoying; the removal of the material solved the problem.

Of the four overcorrections, one achieved a bad result with an angle of 50° and required a posterior fusion. For another, the cable was cut to stop the issue, with a good result (Cobb < 20°) at the end. Of the last two, the result at two years was good as well, and the patients asked for material removal.

Screw issues occurred in four patients: two times the proximal screws broke, and only with the CTJ+™ material from NEUROFRANCE^®^ were there no consequences. The other two times it was the distal screws that came out; a revision was needed to put in a new screw and change the cable.

A curve progression was observed in two patients and led to a posterior fusion. 

## 4. Discussion

The main finding of the present study is that Posterior Vertebral Body Tethering decreases the Cobb angle of the main curve of 70% (from 43.9° to 13.2°) at two years; this is similar to the average correction of the few studies on lumbar Anterior Vertebral Body Tethering: 82% for Pehlivanoglu [16] and 57% for Boeyer [5].

**Figure 7:**
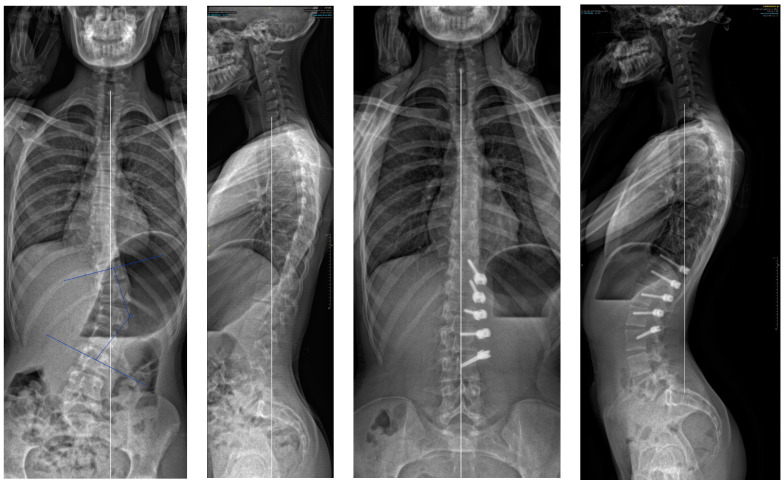
A 13-year-old female, Risser 0. The lumbar curve measured 38° pre-operatively, improved to −10° (slight overcorrection) at two years. In this case, there was an augmentation of both the lumbar lordosis (40° to 58°) and thoracic kyphosis (22° to 37°).

If we analyse the correction and look first at the results after surgery and before, there was an initial improvement of the major curve from 43.9° to 20.3° (54%) due to the “brace effect”, as was observed in other studies [1]. But if we compare the results after surgery and at two years (Figure 7), it seems there was not much correction by growth modulation as described in Anterior Vertebral Body Tethering [1,17,18]. Indeed, there was an amelioration of 7.1° (35%), but it was not statistically significant. This result was unexpected, and must be investigate with studies involving more patients as there was clearly a growth modulation on several cases, leading to an overcorrection. An explanation could be the average old age of the patients with not enough growth remaining. 

The pre-operative bending Cobb angle seems a good tool to predict the outcome of the surgery as there was no difference between the mean pre-operative bending and at two years. It also shows the importance of the flexibility of the spine in vertebral body tethering to achieve the best “brace effect” and so avoid or delay fusion [1,3].

Surprisingly, lumbar lordosis did not change as there was no difference in lumbar angles at two years and pre-operative bending. The fact that the screws are posterior would have suggested an increase in the lumbar lordosis. The thoracic kyphosis did not change, either. On this topic, studies showed a positive or neutral effect of Anterior Vertebral Body Tethering on the thoracic kyphosis [1,6,12,19,20], but we did not find any who evaluate the lumbar lordosis. Further investigations will be necessary to assess and to help understand this result. In any case, this is very interesting to know for the surgical strategy. 

To our knowledge, this is the only study which evaluates lumbar vertebral body tethering using a posterior approach. The anterior approach has been described in a few studies [1,5,12,16]; it is technically demanding, and could lead to nerves issues and severe blood loss. The main operative time described in studies is 3.8 h [8], much longer than Posterior Vertebral Body Tethering, which takes about 2 h. Indeed, the technique is much easier to implement with less risk and a gentle learning curve. Moreover, all spine surgeons are used to the posterior approach, but few perform the anterior approach regularly. The length of stay was 2 to 5 days, similar to Anterior Vertebral Body Tethering [8,21].

The main advantage of vertebral body tethering is to keep spine mobility. A lot of studies demonstrate that this mobility helps to compensate sagittal issues and that a loss of lumbar mobility could lead to functional disability [11,12,16,17,22,23,24,25,26]. The posterior Wiltse approach respects spine mobility as much as an anterior approach.

The complication rate may seem high (59%), but only 13.6% led to a fusion; this is also probably due to the learning curves, as there was no complication for the last eight patients.

Overcorrection occurred in 18%, and is often described as common [1,3] and usually concerning the youngest patients [27]. This shows that both the brace effect and tether effect can be powerful. The brace effect seems more important in lumbar than in thoracic approaches and can lead to an overcorrection. Optimizing surgical timing will help to reduce this complication as there was no overcorrection in patient Risser 3 or higher. This can justify the cutting of the cord (one case).

In the literature, tether breakage has been reported as 2% in lumbar for Courvoisier [1], 50% at 2 years for Pehlivanoglu [16], and 71% for Baroncini [28], who also remarked that a severe and stiff pre-operative curve or a post-operative bad result led to a higher risk of tether breakage.

No tether breakage occurred in this study. The posterior position of the cable, in the main plane of mobility, could be an explanation.

The two screws that pulled out were always on the distal screws. It is very important to put in a screw as big and as long as possible to avoid this issue.

No infections were reported.

**Figure 8:**
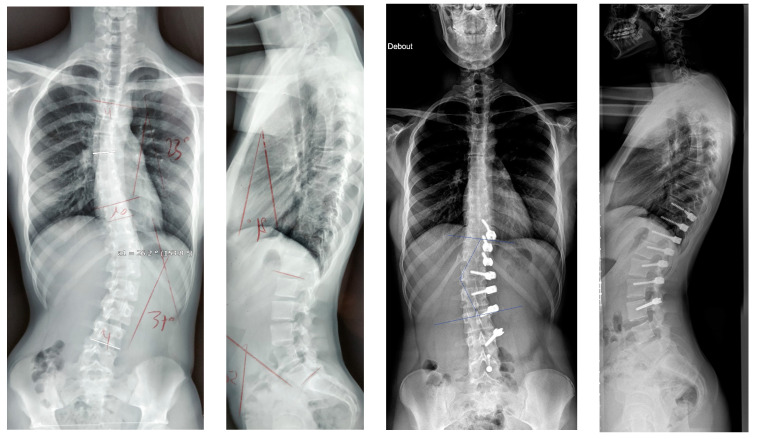
A 13-year-old female, Risser 2. The main curve measured 37° pre-operatively and −18° at 2 years. The overcorrection did not change the good result. In this case the secondary curve improve from 23° to 0°. Note also the broken screw.

The material itself is also important. Screw breakage only occured with the CTJ+™ material from Neurofrance^®^ and always on the proximal screw (Figure 8). Indeed, these screws had a very wide thread and a thin core, making them probably less strong for the same diameter. No breakage occurred with the Braive™ or the Reflect™ screws. Anyway, these breaks did not change the outcome of the concerned patients.

We have seen that screw issues happened at the extremities (proximal and distal). Effectively, these screws are subjected to stress in only one direction, while for the others the forces are balanced on both sides. For this reason, now we suggest adding one more vertebrae proximally and distally to serve as an anchor for the real upper or lower vertebrae and to tighten the cable gently at those levels. For example, if a T11 to L3 correction is necessary, we suggest an instrumentation from T10 to L4 with a gentle tension between T10–T11 and L3–L4.

**Figure 9:**
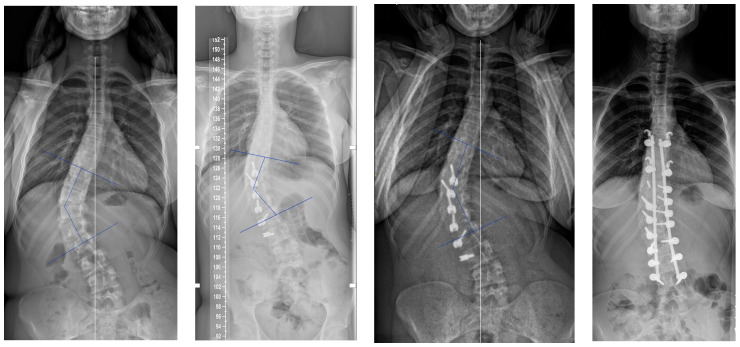
A 15-year-old female, Risser 3 measuring 50° pre-operatively and 44° post-operatively; became worse, requiring a posterior fusion.

As previously mentioned, a new surgery technique with posterior fusion has been necessary in three cases (13.6%); this rate is comparable to other studies [1,29]. In two cases, it was due to a lack of correction (Figure 9) in patients with an initial Cobb angle probably too important (>50°): this seems to be the limit for the Cobb angle for Posterior Vertebral Body Tethering unless the spine is very flexible. The other one was an overcorrection in a patient: Risser 0, Y cartilage open and a very flexible spine who was probably operated on too soon. 

We have found that if the mean angle between pre-operative bending and pre-operative standing Cobb is less than 30°, the outcome will probably be good. For example, a patient with a lumbar Cobb angle of 46° standing and 10° bending should have a good outcome ((46 + 10)/2 = 28 < 30). If this number is more than 40°, the result is less predictable. Currently, the ideal patient would be Risser 2 or more (to avoid hypercorrection) with a mean angle (as described above) under 40.

Material removal was carried out in three cases: one due to pain, and the two others at the will of the patients. We think it is possible (and probably best) to remove the material after the end of growth on all the patients with hypercorrection. In fact, in these cases, the cable has no more effect. But if there is still an angulation, the removal may not be a good idea as a loss of correction could occur after.

## 5. Conclusions

Posterior vertebral body tethering seems a promising technique for the treatment of type 5C lumbar adolescent idiopathic scoliosis. In our view, it is essential to keep as much spine mobility as possible, and for selected patients, it should be discussed as an alternative option before fusion. With experience, we will learn who exactly are these selected patients, and when to operate on them and so increase the efficiency of this technique. The complication rate was high but the issues were easy to resolve and became rare with experience. The new material available also helps a lot. When the technique fails and fusion is necessary, the surgery is not an issue as the spine approach will be similar.

The next step is to evaluate posterior vertebral body tethering combined with thoracic anterior vertebral body tethering for double curves (Lenke 3A or 3C) and will be the subject of another study.

## Figures and Tables

**Table 1:** Mean Cobb angles and sagittal angles (standard deviation).

	Pre-Operative	First Standing	Two Years
Major curve bending	15.6° (8.8)	Not applicable	Not applicable
Major curve	43.9° (9.2)	20.3° (16.2)	13.2° (28.2)
Secondary curve	29.1° (12.6)	21.9° (11.2)	19.9° (13.9)
Kyphosis (T4T12)	23.2° (7.8)	25.1° (9.5)	26.9° (12.6)
Lordosis (L1L5)	41.7° (7.8)	42.4° (10.1)	42.8° (7.5)

**Table 2:** Variation of lumbar Cobb angles.

	Main Curve Improvement in Percentages	Main Curve Improvement in Degrees	*p* Value
2Y to POB	15%	2.4°	0.96
FS to POS	54%	23.6°	0.00000002
2Y to POS	70%	30.7°	0.00005
2Y to FS	35%	7.1°	0.56

**Table 3:** Complications.

	Number of Patients (%)	Treatment	Final Result Consequence
Pain	3 (13.6%)	1 painkiller, physiotherapy 1 screw removed (intra-canal) 1 material remove	none
Overcorrection	4 (18.1%)	1 tether section 1 posterior fusion 2 material remove	1 posterior fusion none for the others
Screw pulled out or screw breakage	4 (18.1%)	2 revisions 2 without consequence	none
Curve progression	2 (9%)	2 posterior fusion	2 posteriors fusions

## Data Availability

Data are contained within the article.
